# Prime Minister’s Overarching Scheme for Holistic Nutrition (POSHAN) on Wheels as a Drive Towards Combating Malnutrition Among Children in Coastal Gujarat

**DOI:** 10.7759/cureus.30137

**Published:** 2022-10-10

**Authors:** Tanveer M Umallawala, Pooja Yadav, Somen Saha, Mayur B Wanjari, Deepak Saxena

**Affiliations:** 1 Epidemiology and Public Health, Indian Institute of Public Health Gandhinagar, Gandhinagar, IND; 2 Public Health, Indian Institute of Public Health Gandhinagar, Gandhinagar, IND; 3 Public Health, Jawaharlal Nehru Medical College, Datta Meghe Institute of Medical Sciences, Wardha, IND; 4 Research, Jawaharlal Nehru Medical College, Datta Meghe Institute of Medical Sciences, Wardha, IND

**Keywords:** gujarat, devbhumi dwarka district, nutrition assistant, energy dense nutrition supplement, severe acute malnutrition (sam)

## Abstract

Background

Gujarat, India, is home to severe acute malnutrition. Wasting in children is associated with a higher risk of death if not treated properly. The present study identified children under five years of age with severe acute malnutrition (SAM). It provided energy-dense nutrition supplement (EDNS) during the rising cases of COVID-19 to treat them as per the guidelines of the government of Gujarat in Devbhumi Dwarka District of Gujarat State, India.

Methods

A descriptive research design was used in the study. Children were screened by a Nutrition Assistant in the presence of an Anganwadi Worker (AWW), Auxiliary Nurse Midwife (ANM)/Community Health Officer (CHO) at Anganwadi Centre or the Child’s home using weight/length Z score <-3 standard deviations (SDs) or mid-upper arm circumference (MUAC) <11.5 and identified severe acute malnourished children were provided EDNS (WHO composition) for a period of seven days initially for a starting period as per the child’s body weight then followed up to eight weeks. Data was entered on the spot in a Google sheet, which nutrition assistants maintained. Data were analyzed using IBM SPSS Statistics for Windows, Version 26.0 (Released 2019; IBM Corp., Armonk, New York, United States) and Microsoft Excel 2019.

Results

The study revealed that 23% of children were considered in the SAM category, followed by 21% in the Bhanvad block and 24% in the Dwarka block. For the Bhanvad block, 40% of the children were treated normally with a maximum weight gain of 1 to 2 kgs (63%). Similarly, for the Dwarka block, 29% of children were treated normally with a weight gain of 1 to 2 kgs (64%).

Conclusions

The study identified children with SAM and provided EDNS for eight weeks. To strengthen the program, the engagement of frontline functionaries of government should be increased, which plays an active role in the community and can be a bridge to the community. As in the community-based management of acute malnutrition (CMAM) program, Accredited Social Health Activists (ASHAs) are responsible for reaching out the ready-to-use therapeutic food (RUTF) to the mothers, weighing of children is done jointly by AWWs and ASHAs on a weekly basis, as well as counselling of the mothers on care and feeding practices and hygiene, and therefore every ASHA receives an incentive of Rs. 25 per child per week to monitor the progress of the child, reach out the therapeutic food, and counsel the mother. This system should be linked with Prime Minister's Overarching Scheme for Holistic Nutrition (POSHAN) on wheels program.

## Introduction

Gujarat, India, is home to severe acute malnutrition (SAM) and is working on several fronts to battle widespread malnutrition among children in the state. SAM is defined by a very low weight-for-height/weight-for-length (wasting) or clinical signs of bilateral pitting oedema or a very low mid-upper arm circumference (MUAC). Children with severe malnutrition are at increased risk of mortality due to common childhood illnesses and reduced immunity and in some cases, a deranged metabolic system. Early identification of SAM is important for initiating treatment and minimizing the risk of complications [1]

Child malnutrition is a significant concern and a dominant cause of death among children in middle-income nations. Malnutrition is coined as a double burden of nutrition by the World Health Organization (WHO). Globally, malnutrition is assessed with indicators such as stunted, wasted, underweight and overweight. Malnutrition has various effects on children aged 1-5 years. It increases the chances of developing anaemia and facing difficulties in education due to mental impairment, physical issues, and susceptibility to non-communicable diseases (specifically, acute respiratory syndrome) [2].

Wasting is categorized as low weight-for-height/weight-for-length. It is physical degradation measured through the loss of muscle and fat tissue. Wasting in children is the result of recent rapid weight loss or failure to gain weight due to hunger and/or disease. Children who are wasted have lower immunity and mental impairments as a result of not reaching developmental milestones and develop oedema formed due to swelling or fluid as an indication of malnutrition and have an increased risk of death [3].

As per the Global Nutrition Report 2021, 149.2 million children are stunted, 45.5 million are wasted, 17 million children suffer from severe wasting, and 20.5 million newborns are born with low birth weight [3]. In India, as per National Family Health Survey-5 data, 35.5% of children below five years were stunted, 19.3% were wasted, 7.7% were severely wasted, and 32.1% were underweight. In Gujarat, 39% of children under the age of 5 years are stunted, 25.1% of children are wasted, 10.6% are severely wasted, and 39.7% of children are underweight. Gujarat has a higher prevalence of wasting and severe wasting than India. There is an increase in the prevalence of severe wasting from National Family Health Survey (NFHS)-4 (2015-16) to NFHS-5 (2019-20) in Gujarat (from 9.5% to 10.6%) [4].

POSHAN, a vernacular term for nutrition, also Prime Minister’s Overarching Scheme for Holistic Nourishment, is a flagship programme to address malnutrition in India. Better nutrition is related to improved infant, child, and maternal health, stronger immune systems, safer pregnancy and childbirth, lower risk of non-communicable diseases (such as diabetes and cardiovascular disease), and longevity [5]. Ready-to-use therapeutic food (RUTF) is an energy-dense, micronutrient-enhanced paste used in therapeutic feeding. For several reasons, RUTF is essential for the community-based management of children who are suffering from uncomplicated SAM and who retain an appetite [6].

In the majority (85-90%) of cases, the malnourished children (or uncomplicated SAM cases) can be looked after at home with the help of community health workers. Under the community-based management of acute malnutrition or CMAM programme, RUTF is being used by the Gujarat government, and being produced and developed by Anand Milk Union Limited (Amul; Anand, India) [7]. The successful implementation of the CMAM programme in Gujarat can serve as a model for India in addressing the problem of malnutrition. RUTFs are energy-dense nutritional supplements (EDNSs). They are an energy-dense, micronutrient-enhanced product used in therapeutic feeding. It provides all the nutrients required for recovery and does not spoil easily even after opening. It has a good shelf life and does not spoil easily even after opening. Since EDNS is not water-based, the risk of bacterial growth is very limited. It is liked by children and is safe and easy to use without close medical supervision. Finally, it can be used in combination with breastfeeding and other best practices for infant and young child feeding. Typical primary ingredients for RUTF include peanuts, oil, sugar, milk powder, and vitamin and mineral supplements [6-8].

CMAM programme in India has achieved low mortality and high cure rates among non-defaulting children. CMAM is an integrated approach that on one hand focuses on treatment and on the other on prevention. A CMAM programme can be an ideal and well-established programme considering the inclusion of different aspects such as alternative feasible solutions, convergent action, multi-stakeholders roles, and accountabilities [8]. Pati et. al in their study conducted in Odisha, India, identified enablers such as a good response from children, village leadership involvement, and multisectoral participation, whereas barriers such as limited awareness, increased workload, and irregular staff payment are linked to programme implementation [9].

Severe acute malnutrition affects an estimated 19 million children under five years of age worldwide and is estimated to account for approximately 400,000 child deaths each year [7]. As per the NFHS-5 data, there are 17.2% of severely wasted children under the age of five years in Devbhumi Dwarka, Gujarat, India [4]. Thus, the purpose of the current study is to screen and identify children under five years of age with severe acute malnutrition at the community level and provide EDNS as per the guidelines of the Government of Gujarat in line with the CMAM programme. It also aims to refer children to higher facilities and assist in providing treatment in case of medical complications.

This approach was planned in case of rising COVID cases and the subsequent second wave of the pandemic with an aim of giving door-to-door services following COVID protocols. This was a unique initiative named “Tushti Poshan Rath” under Project Tushti implemented by the Indian Institute of Public Health Gandhinagar (Gandhinagar, India) jointly with John Snow, Inc. (JSI) Research and Training (R&T) India Foundation (Delhi, India) supported by Nayara Energy Limited (Mumbai, India) and Zilla Panchayat, Devbhumi Dwarka, India. Tushti Poshan Rath was implemented in Bhanvad and Dwarka Blocks of Devbhumi Dwarka District based on the Highest Concentration of SAM children.

## Materials and methods

Study setting

Devbhumi Dwarka consists of four blocks. As per the Devbhumi Dwarka District profile and data collected from Government Department, District Panchayat, Devbhumi Dwarka, it has one district hospital in Khambhaliya block, a sub-district hospital in Dwarka block, 4 Community Health Centres (CHCs), 5 Urban Health Centres (UHCs), 23 Primary Health Centres (PHCs), 169 sub-centres, and 150 Health and Wellness Centres in the district. Like the rest of the state, Accredited Social Health Activists (ASHAs), Auxiliary Nurse Midwives (ANMs) and Anganwadi Workers (AWWs) are present at the village level to provide health, nutrition, education, and related services to young children. A total of 691 Anganwadi Centres (AWCs) at the village level were functional across four blocks during the study period. There are no functional malnutrition treatment centres in the district consisting of four blocks. Two Child Malnutrition Treatment Centres (CMTCs) were developed and made functional under Project Tushti in two blocks on a public-private partnership model.

In this study, a short questionnaire was developed which can also be called a screening tool. The questionnaire consists of the details of the child like Technology Enabled Community Health Operations (TeCHO) ID, name, age, date of birth, birth weight, type of ration card, type of delivery, the child having any birth and medical complications, EDNS packets details like how many packets received, how many packets consumed, and anthropometric details: height, weight, and MUAC for each follow-up.

Intervention design

A descriptive research design was used in the study. Descriptive research is used as a research method that describes the characteristics of the population or phenomenon studied.

In this study, Under Tushti Poshan Rath, nutrition assistant was accompanied along with the necessary equipment for anthropometric measurements such as infantometer, stadiometer, baby weighing scale, bathroom scale, MUAC tape, leaflets, EDNS, etc. Based on the line list of severely underweight children under five years which was obtained from the Integrated Child Development Scheme program Devbhumi Dwarka, the POSHAN rath team organised camps at Anganwadi Centres, where in presence of Anganwadi workers, the team screened the children and identified SAM children and provided EDNS to them weekly for the first time and then followed up tIll eight weeks through counselling and telephone. As per standard protocol, EDNS is provided to the child for a period of six to eight weeks which was followed under the study [6].

An average full course of treatment for a child amounts to around 10-15 kilogrammes of RUTF over a six to eight-week period, which is approximately one carton of RUTF (150 sachets) [6]. Per packet of EDNS contains 92 grams of weight and 500 kcal of energy. The amount of food given to the child should be sufficient to take care of the caloric requirement of 200 kcal per kilogram of body weight per day (200 kcal/kg/day).

SAM child is provided EDNS as per the body weight. The nutrition assistant provided counselling to parents of severely underweight (SUW) children on the children's health, the importance of feeding practices and ways to prepare recipes from Take Home Ration (THR) and locally available ingredients, and key messages (Table 1). During counselling sessions, key messages were explained through a demonstration of hand-washing practices, EDNS packets, and on-spot EDNS feeding to children. Counselling sessions were conducted once when the child was visited for the first time while screening and after every 15 days of physical follow-up.

**Table 1:** Counselling messages for parents of SAM children SAM = severe acute malnutrition; EDNS = energy-dense nutritional supplement

Sr. No.	Key Messages
1.	EDNS is a medicinal food for children with SAM only. It should NOT be shared with any other child.
2.	The mother/caretaker should wash her/his and the child’s hands with soap before preparing the feed and feeding the child respectively.
3.	To give EDNS 6-8 times a day in small amounts.
4.	It should be given to the child after breastfeeding and before any other food.
5.	Sick children often do not like to eat. Give small regular feeds of EDNS and encourage the child to eat often (if possible eight feeds a day).
6.	If the child is breastfed, breastfeed the child before giving EDNS and should be continued.
7.	If the child finishes the recommended amount of EDNS being given and is still hungry, she/he can be given any other foods (supplementary food, local homemade food).
8.	Always offer the child plenty of clean water to drink while he/she is eating the EDNS.
9.	Keep food clean and covered.
10.	When a child has diarrhoea, do not stop feeding. Continue to feed EDNS and (if applicable) breastmilk.
11.	If the child is able to eat on his/her own, then encourage him/her to do so.
12.	To demonstrate the steps of hand washing.

Children’s records are maintained and monitored for the first 24 to 48 hours by telephonic follow-up and counselling sessions and by weekly telephonic follow-up and 15 days of physical follow-up. Follow-ups are continued for a period of six to eight weeks and the child gains weight as per the World Health Organization criteria: weight/length Z score (-1 to +1) SD, MUAC >12.5cm with no medical complications.

In order to prevent any adverse drug reactions (ADRs) after the initial visit, telephonic follow-up with the parents of SAM child,for the first 24 and 48 hours was conducted. If the child was identified with ADR, he/she would be referred to a treatment centre. Weekly telephonic follow-up was conducted with ASHA and AWW for weight and EDNS consumption in children. Physical follow-up was conducted every 15 days by Nutrition Assistants, MUAC and Weight for Height SD scores were measured, and EDNS packets were provided if finished by the child. Once in a fortnight, MUAC and Weight for Height SD scores were measured and children were assessed for any medical complications once a month. During every visit, the child’s weight was monitored.

The child was followed up for a period of about eight weeks whereas in cases where the child did not gain weight, he/she was referred to CMTC as per the guidelines of facility-based management of SAM. Children identified with medical complications were referred to CMTCs at CHC Bhanvad and Sub Divisional Hospital, Dwarka. Medical complications such as neonatal convulsion, syndromic and chromosomal abnormality, early cerebral palsy, and secondary hypoxic-ischemic encephalopathy were observed in children with SAM. Furthermore, the children were mentally retarded and had delayed development. They were advised to be referred to the nearest CMTCs, PHCs/CHCs or private facility if the complications were more severe and also advised for physiotherapy treatment of the child.

After the discharge of the child from POSHAN rath, ASHA and AWW would jointly do follow-up of a child at the community level for a period of two years; it would be ensured that the child was enrolled in Integrated Child Development Scheme (ICDS) and availed of the services of the ICDS scheme.

Selection Criteria

**Figure 1:**
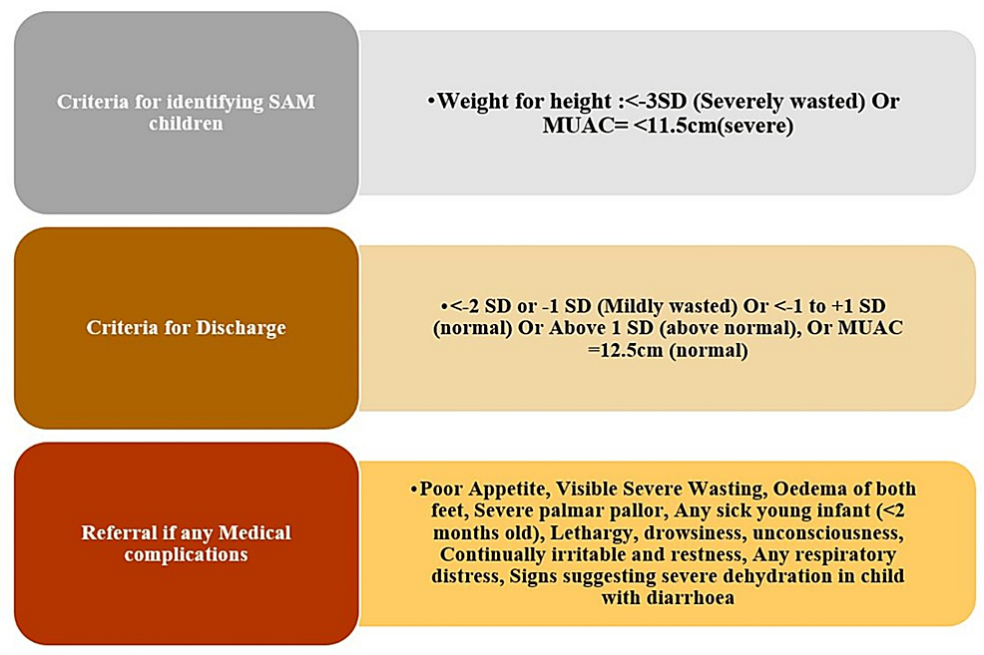
Selection criteria

In this study, a monthly route plan was prepared based on the highest concentration of SAM children in villages. Children were screened by a Nutrition Assistant in presence of an ANM/Community Health Officer (CHO) at Anganwadi Centre or at the child’s home using weight/length Z score <-3 SD or MUAC <11.5 and identified SAM children were provided EDNS (WHO composition) for a period of seven days initially for a starting period as per the child’s body weight. A short questionnaire was developed in English, which was filled out by the Nutrition Assistant and records were maintained of each child for further follow-up. The tool was reviewed by public health nutrition experts, pilot-tested for validity, and modified accordingly. Data was entered on the spot in Google sheets, which were maintained by the Nutrition Assistants. Data were analyzed using IBM SPSS Statistics for Windows, Version 26.0 (Released 2019; IBM Corp., Armonk, New York, United States) and Microsoft Excel 2019 (Figure 1). 

Ethical consideration

For the study purpose, formal approval was obtained from state and district authorities. The Institutional Ethics Committee of the Indian Institute of Public Health, Gandhinagar, approved the study (IEC/IRB approval number 14/2019-20).

## Results

During the period from May 21 to March 22, a total of 556 children were screened covering both Bhanvad and Dwarka Blocks. The majority of the children were in Dwarka Block (57%) whereas 43% were in Bhanvad Block. As per the route plan prepared, all highest concentration villages of SAM children were covered for both blocks (Table 2).

**Table 2:** Block-wise distribution of children

Block	Frequency (N)	Percentage (%)
Bhanvad	240	43.2
Dwarka	315	56.8
Total	555	100.0

Half of the children fell in the age group of 36 to 48 months and above. The mean age group of children was 36 months. There was an equal distribution of males and females in the Bhanvad block, whereas females were more than males in the Dwarka block, and also overall. The majority of children were from the Other Backward Class (OBC) category. This denotes that the majority of the population was an educationally or socio-economically disadvantaged population, which directly links to malnutrition. The population was above the poverty line (70%) (Table 3).

**Table 3:** General information of the study population APL: above poverty line; BPL: below poverty line; NA: not available

Age in completed months	Bhanvad	Dwarka	Total
	N(%)	N(%)	N(%)
0 to 6 m	12(5.0)	2(0.6)	14(2.5)
6 to 12 m	15(6.3)	14(4.4)	29(5.2)
12 to 24 m	27(11.3)	63(20.0)	90(16.2)
24 to 36 m	60(25.0)	75(23.8)	135(24.3)
36 to 48 m	65(27.1)	79(25.1)	144(25.9)
48 & above	61(25.4)	82(26.0)	143(25.8)
Total	240(100)	315(100.0)	555(100)
Mean-35.9 months			
Gender	Bhanvad	Dwarka	Total
Female	120 (50)	172(54.6)	292(52.8)
Male	120 (50)	143(45.4)	263(47.4)
Total	240(100)	315 (100)	555 (100)
Cast	Bhanvad	Dwarka	Total
General	7(2.9)	27(8.6)	34(6.1)
OBC	183(76.25)	245(77.8)	428(77.1)
SC	22(9.2)	24(7.6)	46(8.3)
ST	28(11.7)	19(6.0)	47(8.5)
Total	240(100)	315(100)	555(100)
Type of Ration card	Bhanvad	Dwarka	Total
APL	130(54.2)	259(82.2)	389(70.1)
BPL	107(44.6)	49(15.6)	156(28.1)
NA	3(1.3)	7(2.2)	10 (1.8)
Total	240(100)	315(100)	554(100)

Overall, the majority of the children were delivered under the institution category (96%). Baseline assessment under Project Tushti has highlighted a higher percentage of institutional delivery (99%) which is appreciable, but the concern is the poor uptake of government hospitals, with only 49% of children delivered in them. On the other hand, the use of private healthcare providers indicates a lack of trust in government healthcare services.

About 36% of the children were delivered with low birth weight (<2.5Kg) and 64% were delivered normally (Table 4). In Dwarka, low birth weight delivered is slightly high (4.4%) than in the Bhanvad block. Low birth weight is considered the single most important predictor determinant of infant and childhood morbidity, particularly of neurodevelopment impairments such as mental retardation, learning disability, and infant mortality.

**Table 4:** Risk factors associated with malnutrition

Type of Delivery	Bhanvad	Dwarka	Total
	N (%)	N (%)	N (%)
Full term	230(96.2)	293(93.01)	523(94.4)
Pre-term	9(3.8)	22(6.9)	31(5.6)
Total	239(100)	315(100)	554(100)
Birth weight	Bhanvad	Dwarka	Total
< 2.5kgs- Low birth weight	82(34.3)	122(38.7)	201(36.3)
≥2.5kgs-Normal	157(65.7)	193(61.3)	353(63.7)
Total	240(100)	315(100)	554(100)
Birth Place	Bhanvad	Dwarka	Total
Home delivery	5(2.08)	15(4.8)	20(3.6)
Institutional delivery	235(97.9)	300(95.2)	535(96.4)
Total	240(100)	315(100)	555(100)

The selection criteria for children were as mentioned above. With wasting categorizes as weight and height, overall 48% of the children fell in the normal category, whereas 52% were in the moderate and severe category. Looking into wasting, 19% of children were in the severe category, and 6% were when measured MUAC. As per the criteria of severe acute malnutrition, overall 23% of children were considered SAM followed by 21% in the Bhanvad block and 24 % in the Dwarka block (Table 5). Birth complications are categorized as complications occurred during birth such as hydrocephalus, imperforated anus, anaemia, convulsions during birth, weak digestive system, epilepsy, weak liver, neonatal convulsions, handicapped, birth defects and delayed development. There were 5% of children with complications during birth in Bhanvad and 12% in the Dwarka block. Medical complications which were present during the screening of children were imperforated anus, B12 deficiency, thalassemia, a syndromic child with severe acute malnutrition, epilepsy, early cerebral palsy, ventricular septal defect, small umbilical hernia, phimosis, anaemia, central hypotonia, chromosomal, seizure genetic metabolic disorder, small ostium secundum with atrial septal defect, etc. Dwarka block had a higher percentage of children with medical complications as compared with the Bhanvad block (Table 5).

**Table 5:** Criteria for identifying severe acute malnutrition in children

Weight for height (WHZ)-Wasting	Bhanvad	Dwarka	Total
Moderate	77(32.2)	106(33.8)	183(33.1)
Normal	116(48.5)	149(47.5)	265(47.9)
Severe	46(19.2)	59(18.8)	105(19.0)
Total	239(100)	314(100)	553(100)
Mid-Upper arm circumference	Bhanvad	Dwarka	Total
Moderate	69(28.8)	101(32.1)	170(30.6)
Normal	157(65.4)	194(61.6)	351(63.2)
Severe	11(4.6)	20(6.3)	31(5.6)
Total	240(100)	315(100)	555(100)
Criteria of considering severe acute malnutrition	Bhanvad	Dwarka	Total
MAM-Moderate acute malnutrition	57(23.8)	84(26.7)	141(25.5)
Normal	133(55.6)	154(48.9)	287(51.8)
SAM-Severe acute malnutrition	49(20.5)	77(24.4)	126(22.7)
Total	239(100)	315(100)	554(100)
Any birth complications	Bhanvad	Dwarka	Total
No	227(94.6)	278(88.3)	504(90.8)
Yes	13(5.4)	37(11.7)	51(9.2)
Total	240(100)	315(100)	555(100)
Any medical complications	Bhanvad	Dwarka	Total
No	236(98.3)	284(90.2)	517(93.2)
Yes	4(1.7)	31(9.8)	38(6.8)
Total	240(100)	315(100)	555(100)

**Figure 2:**
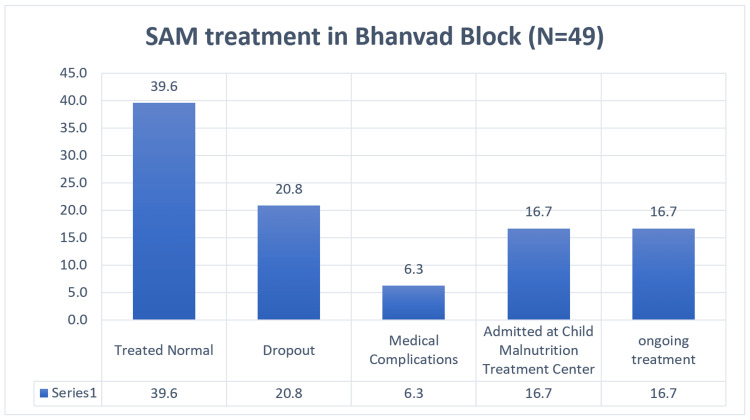
SAM treatment in Bhanvad Block SAM = severe acute malnutrition

**Figure 3:**
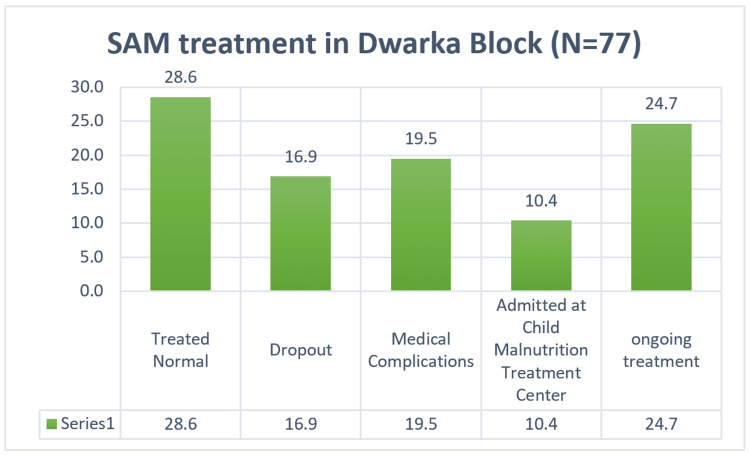
SAM treatment in Dwarka Block SAM = severe acute malnutrition

In the treatment of children (Figure 2) for Bhanvad block, 40% of the children were treated normally with a weight gain of a maximum of 1 to 2 kgs (63%). There were 21% of children who were dropouts for a reason of ADR, diarrhoea, vomiting, taste buds, left the place, etc., which were further moved to a treatment centre, of which 17% were treated. Similarly, for Dwarka block (Figure 3), 29% of children were normal, 17% were dropouts of which 10% were treated at centre. The average weight gain of the children completing treatment was about 1.2 kgs (Table 6).

**Table 6:** Weight gain of children completing treatment

Weight gain in Kgs for children treated	Bhanvad N(%)	Dwarka N(%)
<1 kg	6 (31.6)	7(31.8)
1 to 2 kgs	12 (63.2)	14(63.6)
≥2 kgs and above	1 (5.3)	4(4.5)
Total	19(100)	22(100)

## Discussion

The study was conducted during rising COVID cases and the subsequent second wave of the pandemic with an objective to screen and identify children with severe acute malnutrition at the community level and provide EDNS as per the guidelines of the Government of Gujarat in line with the CMAM programme. This intervention design says that the majority of the children were covered from the Dwarka block (53%) and then the Bhanvad block. Females were more in the Dwarka block as compared to the Bhanvad block. Almost the majority of the children belonged to the OBC category.

There was majority of children delivered with full-term delivery and 64% with ≥2.5kgs birth weight, which is considered to be normal. About 96% of children are delivered under institutions with an indicator of lack of usage of government healthcare services. As per the criteria of severe acute malnutrition, overall, 23% of children were SAM with a majority (24 %) in Dwarka block and then Bhanvad block (21%). Moreover, birth complications and medical complications are majorly seen in Dwarka block as compared to the Bhanvad block.

In the current study, after providing EDNS for a period of eight weeks, 40% of the children were treated normally with a weight gain of a maximum of 1 to 2 kgs (63%) and 29% of children were treated normally with a weight gain of 1 to 2 kgs (64%). Zubaida et al. in her study revealed that 58% of children were cured under the CMAM programme. The study population covered (7,742) was more than in the current study (555) [8]. In a Malawian study, 1,178 children with moderate or severe wasting, kwashiorkor, or both were randomly assigned to receive either home-based RUTF or standard therapy (F-75 and F-100 formulas) and their recovery rates were evaluated. Children who received home-based RUTF had higher rates of weight gain (3.5 vs 2.0 g/kg/day) and had a lower prevalence of fever, cough and diarrhoea. Hence, home-based RUTF was found to have better outcomes in the treatment of malnutrition [9].

Limitations of the study

In this study, the population would migrate and the chances of dropout rise. It was observed that children with SAM had medical complications after birth such as birth asphyxia and after six months of birth they had convulsions, epilepsy, developmental delay, etc. Also, they were born preterm and children with moderate acute malnutrition (MAM) did not have any medical complications. In the case where children had allergic reactions (15%) from EDNS, they were not provided EDNS and were treated at the child malnutrition treatment centres. Also, during follow-ups, sometimes the child was unavailable, which hindered continuous follow-up and tracking of that particular child. Dropouts were marked out with the reasons such as taste-likeliness, cough, adverse drug reactions such as skin rashes, nausea, etc.

## Conclusions

The study identified children with SAM and provided EDNS, which proves to be effective in gaining weight in children. In case of medical complications, the children are treated at the nearest child malnutrition treatment centres and nutritional rehabilitation centres as part of Project Tushti. The community-based malnutrition program needs to be strengthened more with the engagement of frontline functionaries of government who plays an active role in the community and who can be a bridge to the community.
